# Flavonoid Glycosides and Their Derivatives from the Herbs of *Scorzonera austriaca* Wild

**DOI:** 10.3390/molecules21060803

**Published:** 2016-06-21

**Authors:** Yang Xie, Qiu-Shi Guo, Guang-Shu Wang

**Affiliations:** 1 School of Pharmaceutical Sciences, Jilin University, Changchun 130021, China; 13514486691@163.com; 2 Department of Pharmacy, the Second Part of First Hospital, Jilin University, Changchun 130021, China; guoqiushi126@126.com

**Keywords:** *Scorzonera austriaca*, flavonoids, hepatoprotective activities

## Abstract

Five flavonoid glycosides and two derivatives were isolated from the herbs of *Scorzonera austriaca* Wild by silica gel column chromatography and preparative HPLC. Their structures were identified, using chemical and spectroscopic methods, as 5,7,4′-trihydroxyflavone 6-*C*-(2''-*O*-β-d-glucopyranosyl β-d-glucopyranoside) (**1**), 5,7,3′,4′-tetrahydroxyflavone 6-*C*-(2''-*O*-β-d-glucopyranosyl β-d-glucopyranoside) (**2**), quercetin 3-*O*-rutinoside (**3**), 5,7,4′-trihydroxyflavone 6-*C*-β-d-glucopyranoside (**4**), 3′-methoxy-5,7,4′-trihydroxyflavone 6-*C*-β-d-glucopyranoside (**5**), 5,7,4′-trihydroxyflavone 8-*C*-(6''-*O*-trans-caffeoyl β-d-glucopyranoside) (**6**), and 5,7,3′,4′-tetrahydroxyflavone 8-*C*-(6''-*O*-trans-caffeoyl β-d-glucopyranoside) (**7**). Compounds **6** and **7** are new flavonoid glycoside derivatives, and compounds **1**–**5** were isolated from the herbs of *Scorzonera austriaca* for the first time. Compounds **6** and **7** were also assayed for their hepatoprotective activities with rat hepatocytes *in vitro*.

## 1. Introduction

**Figure 1:**
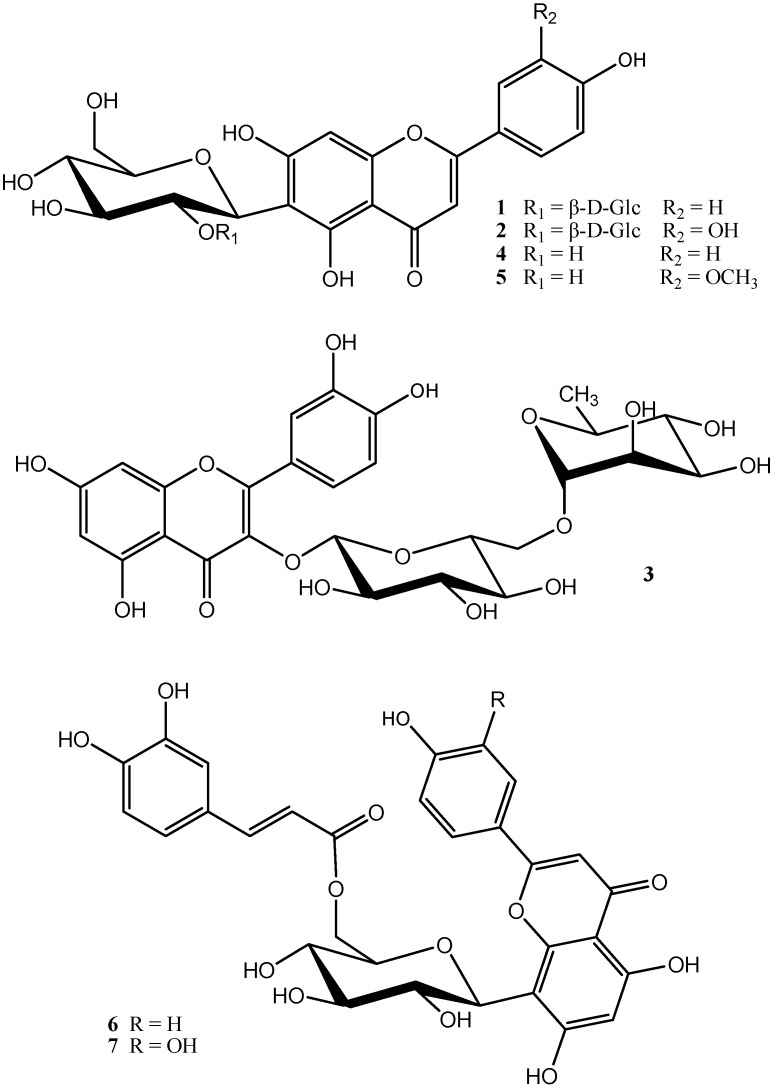
Chemical structures of compounds **1**–**7**.

*Scorzonera austriaca* Wild, a perennial herb of Compositae, is widely distributed in the northeast and northwest regions in China, especially abundant in Jilin Province, and has been widely used for curing fever, carbuncle, and mastitis as a traditional herbal medicine [1]. Since *S. austriaca* is used to treat hepatitis B as a folk medicine in our district, we have investigated its bioactivities and found that the total flavonoids from *S. austriaca* have hepatoprotective effects and inhibitory effects on hepatitis B virus [2,3,4]. Chronic infection with hepatitis B virus (HBV) often leads to the development of liver cancer and cirrhosis, creating immense sociological, clinical, and economic burdens worldwide [5]. Globally, 240 million people are infected with the HBV [6], and 650,000 people die every year from HBV-related cirrhosis or hepatocellular carcinoma [7]. Currently, seven drugs have been approved by FDA for the treatment of HBV infection: two interferons (standard and pegylated) and five nucleoside/nucleotide analogues (lamivudine, adefovir, telbivudine, entecavir, and tenofovir) [8]. Although interferons can restitute the host immune system, and have finite duration and no risk of drug resistance, the need for parenteral administration, the poor long-term response, and the high frequency of adverse side effects make them not ideal [8]. Nucleoside/nucleotide analogues are less expensive and orally available, have minimal side-effects comparing to interferons and can be used for decompensated cirrhosis and after liver transplantation [9]. However, because they have to be taken on a long-term basis, in general, drug resistance may evolve, and there is also a significant risk of HBV reactivation and sometimes HBV flare up after withdrawal of the antiviral agents [10]. Moreover, all of these drugs have low percentage of HBV e antigen seroconversion rate and HBV surface antigen loss, and none of them are able to clear chronic HBV infection [9,11,12]. Thus, new efforts are being directed to develop new and more effective anti-HBV therapeutics. In order to screen the new drug candidate for treating hepatitis B, the isolation and structure identification of flavonoid glycosides and their derivatives from the herbs of *S. austriaca* have been carried out, and we report the isolation and identification of two new flavonoid glycoside derivatives (**6** and **7**), together with five flavonoid glycosides **1**–**5** in the present study (Figure 1).

## 2. Results and Discussion

The air-dried *S. austriaca* herbs were extracted with 70% aqueous ethanol solution (*v*/*v*), the extract was subjected to D101 polyporous resin column chromatography eluated with water and 60% aqueous ethanol solution (*v*/*v*), and the crude flavonoid extracts were obtained from 60% aqueous ethanol eluate. The crude flavonoid extracts were loaded on D4020 polyporous resin column eluting successively with water and different aqueous ethanol solutions, and four fractions were obtained. The four fractions were further separated and purified by silica gel column chromatography and semi-preparative RP-HPLC to afford compounds **1**–**7**, including two new flavonoid glycoside derivatives (**6** and **7**). 

The five known compounds were identified as 5,7,4′-trihydroxyflavone 6-*C*-(2''-*O*-β-d-glucopyranosyl β-d-glucopyranoside) (**1**) [13], 5,7,3′,4′-tetrahydroxyflavone 6-*C*-(2''-*O*-β-d-glucopyranosyl β-d-glucopyranoside) (**2**) [14], quercetin 3-*O*-rutinoside (**3**) [15], 5,7,4′-trihydroxyflavone 6-*C*-β-d-glucopyranoside (**4**) [16], and 3′-methoxy-5,7,4′-trihydroxyflavone 6-*C*-β-d-glucopyranoside (**5**) [17], by comparison of various data with the reported compounds.

**Figure 2:**
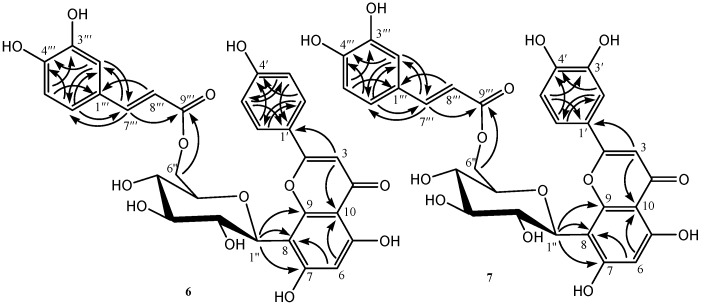
The key HMBC correlation of compounds **6** and **7** (arrows point from proton to carbon).

Compound **6** was obtained as yellow amorphous powders. Its HRESIMS displayed a [M + H]^+^ ion peak at *m*/*z* 595.1437 (calcd for C_30_H_27_O_13_, 595.1446), indicating the molecular formula C_30_H_26_O_13_. The ^1^H- and ^13^C-NMR spectra showed eleven aromatic or unsaturated proton signals, twenty-four unsaturated carbon signals, and six saturated carbons signals, among which a typical three-proton ABX aromatic spin system at δ_H_ 6.70 (1H, d, *J* = 7.0 Hz), 6.91 (1H, d, *J* = 7.0 Hz), 6.80 (1H, br. s), a trans-disubstituted double bond conjugated with a carbonyl group at δ_H_ 7.40 (1H, d, *J* = 15.7 Hz), 6.15 (1H, d, *J* = 15.7 Hz), and nine unsaturated carbon signals at δ_C_ 166.7 (C), 148.4 (C), 145.5 (C), 145.4 (CH), 125.3(C), 120.5 (CH) 115.7 (CH), 115.3 (CH), and 113.6 (CH) were deduced to arise from a trans-caffeoyl moiety based on the analysis of ^1^H-^1^H COSY, HMQC, and HMBC of **6** and the comparison of ^1^H- and ^13^C-NMR spectra of **6** with the reported values [18]. The ^1^H- and ^13^C-NMR spectra of **6** were very similar to those of the reported compound, 5,7,4′-trihydroxyflavone 8-*C*-β-d-glucopyranoside (**6′**) [19], except for a set of signals of the above trans-caffeoyl moiety. Comparison of ^1^H- and ^13^C-NMR spectra data of **6** and **6′** showed that the trans-caffeoyl moiety is attached to C-6″ of glucose on the basis of the acylation effects at the OH of C-6″, which are the downfield shift of the C-6″ protons (+0.66 and +0.73 ppm) and of the C-6″ carbon (+2.7 ppm) and the upfield shift of the C-5″ carbon (−3.5 ppm) [20], which was also confirmed by the correlation of the C-6″ protons at δ_H_ 4.17 and 4.48 with the carboxy carbon at δ_C_ 166.7 in HMBC of **6**. The glucosyl anomeric proton observed at δ_H_ 4.77 as a doublet with *J* = 9.9 Hz was indicative of a β-configuration for the glucose. The full assignments of all protons and carbons were preformed through the correlations in 2D-NMR spectra (^1^H-^1^H COSY, HMQC and HMBC) of **6**. For all of the data of ^1^H-, ^13^C-, and HMBC-NMR of compound **6** see Table 1, and key correlations and the structure of compound **6**, see Figure 2. Based on the above evidence, the structure of **6** was determined to be 5,7,4′-trihydroxyflavone 8-*C*-(6''-*O*-trans-caffeoyl β-d-glucopyranoside). 

Compound **7** was obtained as yellow amorphous powders. Its HRESIMS displayed a [M + H]^+^ ion peak at *m*/*z* 611.1408 (calcd for C_30_H_27_O_14_, 611.1395), indicating the molecular formula C_30_H_26_O_14_. The ^1^H- and ^13^C-NMR spectra of **7** were very similar to those of the reported compound, 5,7,3′,4′-tetrahydroxyflavone 8-*C-*β*-*d-glucopyranoside (**7′**) [21], except for a set of signals indicative of a trans-caffeoyl moiety: δ_H_ 7.40 (1H, d, *J* = 15.8 Hz, H-7″′), 6.23 (1H, d, *J* = 15.8 Hz, H-8″′), 6.94 (1H, s, H-2″′), 6.73 (1H, d, *J* = 8.0 Hz, H-5″′), and 6.85 (1H, d, *J* = 8.0 Hz, H-6″′), and δ_C_ 166.8 (C-9″′), 148.3 (C-4″′), 145.3 (C-3″′, 7″′), 125.4 (C-1″′), 120.6 (C-6″′) 115.8 (C-5″′), 115.6 (C-2″′) and 113.7 (C-8″′). The attachment of the trans-caffeoyl moiety to the 6"-position of the glucosyl part was also deduced from the acylation effects at the OH of C-6″, which are the downfield shift of the C-6″ protons (+0.59 and +0.75 ppm) and of the C-6″ carbon (+2.6 ppm) and the upfield shift of the C-5″ carbon (−3.5 ppm) [19], which was further confirmed by the correlation of the C-6″ protons at δ_H_ 4.15 and 4.54 with the carboxy carbon at δ_C_ 166.8 in HMBC of **7**. The glucosyl anomeric proton observed at δ_H_ 4.75 as a doublet with *J* = 9.9 Hz was indicative of a β-configuration for the glucose. The full assignments of all protons and carbons were preformed through the correlations in 2D-NMR spectra (^1^H-^1^H COSY, HMQC, and HMBC) of **7**. For all of the data of ^1^H-, ^13^C-, and HMBC-NMR of compound **7** see Table 1, and key correlations and the structure of compound **7**, see Figure 2. Based on the above evidence, the structure of **7** was determined to be 5,7,3′,4′-tetrahydroxyflavone 8-*C*-(6''-*O*-trans-caffeoyl β*-*d-glucopyranoside).

The hepatoprotective activities of compounds **6** and **7** were assessed by measuring the content of alanine aminotransferase (ALT) of the cultures of rat hepatocytes injured by CCl_4_. Compounds **6** and **7** exhibited hepatoprotective activities with values of 71.2% and 81.2%, respectively, at a concentration of 100 μM, comparable to that of silibinin [22], which was used as a positive control (68.3% at 50 μM) (Table 2). Therefore, the flavonoid glycoside derivatives **6** and **7** are considered to be two of the hepatoprotective principles in this plant.

## 3. Experimental Section 

### 3.1. General Information 

NMR spectra were recorded on a Bruker AV-400 spectrometer (Bruke Corporation, Faellanden, Switzerland). UV spectra were recorded on a Shimadzu UV-2401A spectrometer (Shimadzu Corporation, Kyoto, Japan). HR-ESI-MS were recorded on a Bruker microOTOF-Q II mass spectrometer (Bruke Corporation, Bremen, Germany). HPLC was performed Shimadzu LC-10A with a SPD-10A detector (Shimadzu Corporation, Kyoto, Japan) and Gemini 5μ C18 110A column (250 mm × 10.00 mm, 5 μm, flow rate: 3.0 mL**·**min^−1^, Phenomenex, Torrance, CA, USA). Column chromatography was performed on silica gel (200–300 mesh, Qingdao Marine Chemical Inc., Qingdao, China), and D101 polyporous resin (Tianjin Pesticide Co., LTD., Resin Branch, Tianjin, China). Thin layer Chromatography was performed on glass precoated silica gel GF_254_ plates (Qingdao Haiyang Chemical Co., Ltd, Qingdao, China), detection under UV light or by spraying with 10% H_2_SO_4_ in 95% EtOH followed by heating. Distilled water was purchased from Hangzhou Wahaha Group Co., Ltd. (Hongzhou, China). Acetonitrile of chromatographic grade for HPLC was purchased from Fisher Scientific (Fair Lawn, NJ, USA). Other chemicals and reagents of analytical grade were from Beijing Chemical Works (Beijing, China).

The bioactivities were measured on a DNM-9602 enzyme immunoassay spectrophotometer (Beijing, China). Enzyme-linked immunosorbent assay kits for alanine transferase (ALT) were purchased from Jiancheng Institute of Biotechnology (Nanjing, China), collagenase IV from Sigma (St. Louis, MO, USA), 1640 medium from HyClone (Logan, UT, USA), PBS from Gibco company (Carlsbad, CA, USA), fetal bovine serum (FBS) from Zhejiang Tianhang Biotechnology Co., Ltd. (Hangzhou, China), and silibinin from the Chinese National Institute for the Control of Pharmaceutical and Biological Products (Beijing, China).

Female Wistar rats (150–180g) were purchased from the Experimental Animal Center, Jilin University (Changchun, China).

The herbs of *S. austriaca* were collected in Si-ping District in Jilin Province, China. They were identified by Professor Jing-Min Zhang of the School of Pharmaceutical Sciences, Jilin University (Changchun, China).

### 3.2. Extraction and Isolation

Two kilograms of air-dried whole *S. austriaca* herbs were extracted twice with 20 L of 70% aqueous ethanol solution (*v*/*v*) at room temperature. The extraction solution was concentrated under reduced pressure to remove ethanol, and the water concentrate was filtered and then passed through a D101 polyporous resin column eluting successively with water and 60% aqueous ethanol solution (*v*/*v*). The crude flavonoid extracts were obtained from 60% aqueous ethanol eluate by vacuum distillation recovery and used for the next experiments. The crude flavonoid extracts were loaded on D4020 polyporous resin column eluting successively with water, 15%, 20%, 25%, 30%, and 40% aqueous ethanol solutions (*v*/*v*), and six fractions (Fractions 1–6) were obtained. Fraction 2 was chromatographed over silica gel eluting with CHCl_3_–MeOH–EtOAc–H_2_O (2:2:4:1, *v*/*v*, lower layer) to afford Fraction 2a, Fraction 4 with CHCl_3_–MeOH–EtOAc–H_2_O (2:1.4:4:1, *v*/*v*, lower layer) to afford Fraction 4a, and Fraction 6 with CHCl_3_–MeOH–EtOAc–H_2_O (2:1:4:1, *v*/*v*, lower layer) to afford Fraction 6a. They were further isolated by semi-preparative RP-HPLC using acetonitrile and 0.1% formic acid solution in water as the mobile phase and the eluate was monitored at 337 nm. Compound **1** (30 mg), **2** (30 mg), and **3** (20 mg) were obtained from Fraction 2a with gradient elution (10%–11% acetonitrile from 0.00–20.00 min, 11%–18% acetonitrile from 20.00–40.00 min), compound **4** (30 mg) and **5** (20 mg) from Fraction 4a by using 15% acetonitrile, and compound **6** (60 mg) and **7** (200 mg) from Fraction 6a with gradient elution (20%–22% acetonitrile from 0.00–45.00 min). 

Compound **1**: Yellow amorphous powder, yielded a positive reaction to FeCl_3_ reagent, mp 201–203 °C; UV (MeOH), λ_max_ 265, 339 nm; HRESIMS *m*/*z* 595.1665 [M + H]^+^ (calcd for C_27_H_31_O_15_, 595.1657); ^1^H-NMR (DMSO-*d*_6_, 400 MHz) δ: 7.93 (2H, d, *J* = 8.4 Hz, H-2′,6′), 6.93 (2H, d, *J* = 8.4 Hz, H-3′,5′), 6.78 (1H, s, H-3), 6.49 (1H, s, H-8), 4.65 (1H, d, *J* = 9.6Hz, Glc-H-1), 4.44 (1H, m, Glc-H-2), 3.41 (1H, m, Glc-H-3), 3.16 (1H, m, Glc-H-4), 3.16 (1H, m, Glc-H-5), 3.33 (1H, m, Glc-H-6), 3.67 (1H, m, Glc-H-6), 4.18 (1H, d, *J* = 7.6Hz, Glc′-H-1), 2.85 (1H, m, Glc′-H-2), 3.03 (1H, m, Glc′-H-3), 3.01 (1H, m, Glc′-H-4), 2.64 (1H, m, Glc′-H-5), 2.94 (1H, m, Glc′-H-6), 3.17 (1H, m, Glc′-H-6); ^13^C-NMR (DMSO-*d*_6_, 100 MHz) δ: 163.4 (C-2), 102.7 (C-3), 182.0 (C-4), 161.1 (C-5), 107.9 (C-6), 161.1 (C-7), 93.3 (C-8), 156.4 (C-9), 103.3 (C-10), 121.1 (C-1′), 128.4 (C-2′,6′), 116.0 (C-3′,5′), 161.1 (C-4′), 71.1 (Glc-1), 81.0 (Glc-2), 78.4 (Glc-3), 70.4 (Glc-4), 81.6 (Glc-5), 61.4 (Glc-6), 105.3 (Glc′-1), 74.7 (Glc′-2), 76.4 (Glc′-3), 69.3 (Glc′-4), 76.4 (Glc′-5), 60.5 (Glc′-6).

Compound **2**: Yellow amorphous powder, yielded a positive reaction to FeCl_3_ reagent, mp 205–207 °C; UV (MeOH), λ_max_ 258, 366 nm; HRESIMS *m*/*z* 611.1623 [M + H]^+^ (calcd for C_27_H_31_O_16_, 611.1607); ^1^H-NMR (DMSO-*d*_6_, 400 MHz) δ: 7.41 (1H, d, *J* = 8.1 Hz, H-6′), 7.40 (H, s, H-2′), 6.89 (1H, d, *J* = 8.1 Hz, H-5′), 6.65 (1H, s, H-3), 6.45 (1H, s, H-8), 4.66 (1H, d, *J* = 9.8Hz, Glc-H-1), 4.44 (1H, m, Glc-H-2), 3.45 (1H, m, Glc-H-3), 3.17 (1H, m, Glc-H-4), 3.17 (1H, m, Glc-H-5), 3.41 (1H, m, Glc-H-6), 3.68 (1H, m, Glc-H-6), 4.19 (1H, d, *J* = 7.6Hz, Glc′-H-1), 2.86 (1H, m, Glc′-H-2), 3.06 (1H, m, Glc′-H-3), 3.03 (1H, m, Glc′-H-4), 2.65 (1H, m, Glc′-H-5), 2.99 (1H, m, Glc′-H-6), 3.17 (1H, m, Glc′-H-6); ^13^C-NMR (DMSO-*d*_6_, 100 MHz) δ: 163.5 (C-2), 102.7 (C-3), 181.9 (C-4), 160.6 (C-5), 107.9 (C-6), 163.0 (C-7), 93.3 (C-8), 156.4 (C-9), 103.3 (C-10), 121.4 (C-1′), 113.2 (C-2′), 145.7 (C-3′), 149.7 (C-4′), 116.0 (C-5′), 118.9 (C-6′), 71.2 (Glc-1), 80.9 (Glc-2), 78.4 (Glc-3), 70.4 (Glc-4), 81.6 (Glc-5), 61.4 (Glc-6), 105.5 (Glc′-1), 74.7 (Glc′-2), 76.2 (Glc′-3′), 69.3 (Glc′-4), 76.2 (Glc′-5), 60.6 (Glc′-6).

Compound **3**: Yellow amorphous powder, yielded a positive reaction to FeCl_3_ reagent, mp 185–187 °C; UV (MeOH), λ_max_ 259, 359 nm; HRESIMS *m*/*z* 611.1620 [M + H]^+^ (calcd for C_27_H_31_O_16_, 611.1607). ^1^H-NMR (DMSO-*d*_6_, 400MHz) δ: 7.54 (1H, d, *J* = 8.1, H-6′), 7.53 (1H, s H-2′), 6.83 (1H, d, *J* = 8.1 Hz, H-5′), 6.36 (1H, s, H-8), 6.17 (1H, s, H-6), 5.33 (1H, d, *J* = 6.7 Hz, Glc-H-1), 4.39 (1H, s, Rha-H-1), 0.99 (3H, d, *J* = 6.1 Hz, Rha-H-6); ^13^C-NMR (DMSO-*d*_6_, 100 MHz) δ: 156.4 (C-2), 133.2 (C-3), 177.2 (C-4), 161.1 (C-5), 98.8 (C-6), 164.7 (C-7), 93.6 (C-8), 156.4 (C-9), 103.6 (C-10), 121.0 (C-1′), 115.2 (C-2′), 144.8 (C-3′), 148.6 (C-4′), 116.1 (C-5′), 121.5 (C-6′), 101.3 (Glc-1), 74.0 (Glc-2), 76.4 (Glc-3), 69.9 (Glc-4), 75.9 (Glc-5), 66.9 (Glc-6), 100.7 (Rha-1), 70.5 (Rha-2), 70.3 (Rha-3), 71.8 (Rha-4), 68.2 (Rha-5), 17.7 (Rha-6).

Compound **4**: Yellow amorphous powder, yielded a positive reaction to FeCl_3_ reagent, mp 238–240 °C; UV (MeOH), λ_max_ 260, 351 nm; HRESIMS *m*/*z* 433.1157 [M + H]^+^ (calcd for C_21_H_21_O_10_, 433.1129); ^1^H-NMR (DMSO-*d*_6_, 400 MHz) δ: 7.90 (2H, d, *J* = 8.4 Hz, H-2′,6′), 6.97 (2H, d, *J* = 8.4 Hz, H-3′,5′), 6.74 (1H, s, H-3), 6.64 (1H, s, H-8), 4.58 (1H, d, *J* = 9.6Hz, Glc-H-1), 4.07 (1H, t, *J* = 8.8Hz, Glc-H-2), 3.20 (1H, m, Glc-H-3), 3.15 (1H, m, Glc-H-4), 3.15 (1H, m, Glc-H-5), 3.43(1H, m, Glc-H-6), 3.67 (1H, d, *J* = 15.6 Hz, Glc-H-6); ^13^C-NMR (DMSO-*d*_6_, 100 MHz) δ: 163.4 (C-2), 102.6 (C-3), 181.8 (C-4), 160.6 (C-5), 108.9 (C-6), 164.0 (C-7), 93.8 (C-8), 156.2 (C-9), 103.1 (C-10), 121.0 (C-1′), 128.3 (C-2′,6′), 116.1 (C-3′,5′), 161.4 (C-4′), 73.0 (Glc-1), 70.1 (Glc-2), 79.0 (Glc-3), 70.5 (Glc-4), 81.5 (Glc-5), 61.3 (Glc-6). 

Compound **5**: Yellow amorphous powder, yielded a positive reaction to FeCl_3_ reagent, mp 208–210 °C; UV (MeOH), λ_max_ 261, 348 nm; HRESIMS *m*/*z* 463.1253 [M + H]^+^ (calcd for C_2__2_H_2__3_O_1__1_, 463.1235); ^1^H-NMR (DMSO-*d*_6_, 400 MHz) δ: 7.33 (1H, d, *J* = 8.4 Hz, H-6′), 7.33 (H, s, H-2′), 6.82 (1H, d, *J* = 8.4 Hz, H-5′), 6.50 (1H, s, H-3), 6.37 (1H, s, H-8), 4.66 (1H, d, *J* = 9.8 Hz, Glc-H-1), 3.25 (3H, s, C-3′-OCH_3_); ^13^C-NMR (DMSO-*d*_6_, 100 MHz) δ: 164.1 (C-2), 102.3 (C-3), 182.0 (C-4), 160.1 (C-5), 107.3 (C-6), 163.4 (C-7), 93.5 (C-8), 156.8 (C-9), 103.2 (C-10), 121.6 (C-1′), 108.6 (C-2′), 147.6 (C-3′), 150.3 (C-4′), 115.1 (C-5′), 120.0 (C-6′), 73.5 (Glc-1), 69.8 (Glc-2), 78.2 (Glc-3), 70.7 (Glc-4), 80.7 (Glc-5), 61.1 (Glc-6).

Compound **6**: Yellow amorphous powder, yielded a positive reaction to FeCl_3_ reagent, mp 208–210 °C; UV (MeOH), λ_max_ 268, 339 nm; HRESIMS *m*/*z* 595.1437 [M + H]^+^ (calcd for C_30_H_27_O_13_, 595.1446); ^1^H-NMR (DMSO-*d*_6_, 400 MHz) and ^13^C-NMR (DMSO-*d*_6_, 100 MHz) see Table 1.

Compound **7**: Yellow amorphous powder, yielded a positive reaction to FeCl_3_ reagent, mp 204–206 °C; UV (MeOH), λ_max_ 269, 350 nm; HRESIMS *m*/*z* 611.1408 [M + H]^+^ (calcd for C_30_H_27_O_14_, 611.1395); ^1^H-NMR (DMSO-*d*_6_, 400 MHz) and ^13^C-NMR (DMSO-*d*_6_, 100 MHz) see Table 1.

### 3.3. In Vitro Hepatoprotective Activity 

Isolated rat hepatocytes from female Wistar rats were prepared by the collagenase perfusion technique with minor modifications [23]. Culture medium was composed of RPMI 1640 medium supplemented with 10% fetal bovine serum and 1% Pen/Strep (100 IU**·**mL^−1^ penicillin and 100 mg**·**mL^−1^ streptomycin). The isolated cells were diluted to 8 × 10^6^ / mL using the culture medium, and every 8 × 10^5^ cells (0.1 mL) were seeded into a 48-well plate and incubated at 37 °C in a humidified atmosphere containing 5% CO_2_. After preincubation for four hours, the medium was replaced with fresh medium containing CCl_4_ (15 mM) and test specimens at various concentrations, and four hours later, the medium was taken to measure ALT. Enzyme-linked immunosorbent assay kits and DNM-9602 enzyme immunoassay spectrophotometer were used to measure ALT. For the results, see the Table 2. 

## 4. Conclusions

Compounds **6** and **7** are new flavonoid glycoside derivatives. Compounds **1**–**5** were isolated from *S. austriaca* Wild for the first time. Compounds **6** and **7** were also assayed for hepatoprotective activities with rat hepatocytes, and the data proved that compounds **6** and **7** exhibited hepatoprotective activities. Therefore, the new flavonoid glycoside derivatives **6** and **7** are considered to be two of the hepatoprotective properties in this plant. 

## Figures and Tables

**Table 1:** ^1^H-NMR (DMSO-*d*_6_, 400 MHz), ^13^C-NMR (DMSO-*d*_6_, 100 MHz), and HMBC data of compounds **6** and **7** (TMS as the internal standard, δ in ppm, *J* in Hz).

No.	6			6′		7			7′	
	δ_C_	δ_H_ *J* (Hz)	HMBC (H→C)	δ_C_	δ_H_ *J* (Hz)	δ_C_	δ_H_ *J* (Hz)	HMBC (H→C)	δ_C_	δ_H_ *J* (Hz)
Aglycone moiety										
2	163.6			163.9		163.9			164.2	
3	102.3	6.80 (1H, s)	103.9, 121.2	102.4	6.77 (1H, s)	102.4	6.68 (1H, s)	104.1, 121.8	102.5	6.64 (1H, s)
4	182.0			182.0		182.0			182.1	
5	161.7			161.1		160.6			160.5	
6	98.2	6.28 (1H, s)	103.9, 104.2	98.1	6.27 (1H, s)	98.1	6.29 (1H, s)	104.1	98.2	6.27 (1H, s)
7	163.0			162.7		162.6			162.6	
8	104.2			104.6		104.1			104.6	
9	160.5			160.4		156.0			156.1	
10	103.9			103.9		104.1			104.1	
1′	121.2			121.6		121.8			122.1	
2′	128.6	8.01 (1H, d, *J* = 7.2 Hz)	128.6, 156.0, 163.6	128.9	8.02 (1H, d, *J* = 7.9 Hz)	113.9	7.48 (1H, s)	119.0, 150.0, 163.9	114.2	7.48 (1H, s)
3′	116.0	6.95 (1H, d, *J* = 7.2 Hz)	121.2, 116.0	115.8	6.89 (1H, d, *J* = 7.9 Hz)	146.0			145.9	
4′	156.0			156.0		150.0			149.7	
5′	116.0	6.95 (1H, d, *J* = 7.2 Hz)	121.2, 116.0	115.8	6.89 (1H, d, *J* = 7.9 Hz)	115.6	6.94 (1H, d, *J* = 8.1 Hz)	121.8, 146.0	115.8	6.86 (1H, d, *J* = 8.3 Hz)
6′	128.6	8.01 (1H, d, *J* = 7.2 Hz)	128.6, 156.0, 163.6	128.9	8.02 (1H, d, *J* = 7.9 Hz)	119.0	7.53 (1H, d, *J* = 8.1 Hz)	113.9, 150.0, 163.9	119.5	7.53 (1H, d, *J* = 8.3 Hz)
Sugar moiety										
1″	73.6	4.77 (1H, d, *J* = 9.9 Hz)	70.7, 78.4, 104.2, 160.5, 163.0	73.4	4.79 (1H, d, *J* = 9.6 Hz)	73.6	4.75 (1H, d, *J* = 9.9 Hz)	70.6, 78.5, 104.1, 156.0, 162.6	73.5	4.68 (1H, d, *J* = 9.4 Hz)
2″	70.7	3.95 (1H, m)		70.8	3.84 (1H, m)	70.6	3.93 (1H, m)		70.9	3.84 (1H, m)
3″	78.4	3.32 (1H, m)		78.6	3.26 (1H, m)	78.5	3.31 (1H, m)		78.8	3.31 (1H, m)
4″	70.5	3.51 (1H, m)		70.5	3.36 (1H, m)	70.5	3.53 (1H, m)		70.8	3.53 (1H, m)
5″	78.3	3.51 (1H, m)		81.8	3.36 (1H, m)	78.5	3.54 (1H, m)		82.0	3.54 (1H, m)
6″	64.0	4.17 (1H, m), 4.48 (1H, m)	166.7	61.3	3.51 (1H, m), 3.75 (1H, m)	64.3	4.15 (1H, m), 4.54 (1H, m)	166.8	61.7	3.56 (1H, m), 3.79 (1H, m)
Caffeoyl moiety										
1″′	125.3					125.4				
2″′	115.5	6.80 (1H, s)	145.4, 148.4, 120.5			115.6	6.94 (1H, s)	145.3, 148.3, 120.6		
3″′	145.5					145.3				
4″′	148.4					148.3				
5″′	115.7	6.70 (1H, d, *J* = 7.0 Hz)	125.3, 145.5			115.8	6.73 (1H, d, *J* = 8.0 Hz)	125.4, 145.3		
6″′	120.5	6.91 (1H, d, *J* = 7.0 Hz)	115.5, 148.4, 145.4			120.6	6.85 (1H, d, *J* = 8.0 Hz)	115.6, 148.3, 145.3		
7″′	145.4	7.40 (1H, d, *J* = 15.7 Hz)	115.5, 120.5, 166.7			145.3	7.40 (1H, d, *J* = 15.8 Hz)	115.6, 120.6, 166.8		
8″′	113.6	6.15 (1H, d, *J* = 15.7 Hz)	125.3			113.7	6.23 (1H, d, *J* = 15.8 Hz)	125.4		
9″′	166.7					166.8				

Note: the assignments were based on DEPT, HMQC, ^1^H-^1^H COSY, and HMBC experiments.

**Table 2:** Effects of compounds **6** and **7** on CCl_4_-induced toxicity of rat hepatocytes.

Group	Dose	ALT (IU/L)	Relative Protection(%)
Control		12.6 ± 2.4	100
CCl_4_-treated		101.5 ± 4.5 *	0
Silibinin	50 μM	40.8 ± 2.9 ^##^	68.3
6	25 μM	86.7 ± 3.6 ^#^	16.5
	50 μM	54.5 ± 3.7 ^##^	52.9
	100 μM	38.2 ± 3.8 ^##^	71.2
7	25 μM	76.6 ± 3.5 ^##^	28.1
	50 μM	42.5 ± 2.4 ^##^	66.4
	100 μM	28.8 ± 3.5 ^##^	81.2

All data were analyzed using SPSS version 20.0 (International Business Machines Corporation, Armonk, NY, USA); the each value represents the mean ± SD (*n* = 3); the % of protection is calculated as 100 × (values of CCl_4_ − value of sample)/(value of CCl_4_ − value of control); * *p* < 0.01, compared with control group; ^#^ *p* < 0.05, ^##^ *p* < 0.01, compared with CCl_4_-treated group.
